# A novel data fusion method for the effective analysis of multiple panels of flow cytometry data

**DOI:** 10.1038/s41598-019-43166-x

**Published:** 2019-05-01

**Authors:** Gerjen H. Tinnevelt, Selma van Staveren, Kristiaan Wouters, Erwin Wijnands, Kenneth Verboven, Rita Folcarelli, Leo Koenderman, Lutgarde M. C. Buydens, Jeroen J. Jansen

**Affiliations:** 1 Radboud University, Institute for Molecules and Materials (Analytical Chemistry), postvak 61, P.O. Box 9010, 6500 GL Nijmegen, The Netherlands; 2 TI-COAST, Science Park 904, 1098 XH Amsterdam, The Netherlands; 3 Department of Respiratory Medicine and laboratory of translational immunology (LTI), University Medical Center Utrecht, Heidelberglaan 100, 3584CX Utrecht, The Netherlands; 4 Deptartment of Internal Medicine Laboratory of Metabolism and Vascular Medicine, P.O. Box 616 (UNS50/14), 6200 MD Maastricht, The Netherlands; 5 Experimental Vascular Pathology group, P.O. Box 5800, 6202 MZ Maastricht, The Netherlands; 6 REVAL - Rehabilitation Research Center, Faculty of Rehabilitation Sciences, Hasselt University, Diepenbeek, Belgium; 7 BIOMED - Biomedical Research Institute, Faculty of Medicine and Life Sciences, Hasselt University, Diepenbeek, Belgium

**Keywords:** Computational models, Predictive markers

## Abstract

Multicolour flow cytometry (MFC) is used to measure multiple cellular markers at the single-cell level. Cellular markers may be coloured with different panels of fluorescently-labelled antibodies to enable cell identification or the detection of activated cells in pre-defined, ‘gated’ specific cell subsets. The number of markers that can be used per measurement is technologically limited however, requiring every panel to be analysed in a separate aliquot measurement. The combined analyses of these dedicated panels may enhance the predictive ability of these measurements and could enrich the interpretation of the immunological information. Here we introduce a fusion method for MFC data, based on DAMACY (Discriminant Analysis of Multi-Aspect Cytometry data), which can combine information from complementary panels. This approach leads to both enhanced predictions and clearer interpretations in comparison with the analysis of separate measurements. We illustrate this method using two datasets: the response of neutrophils evoked by a systemic endotoxin challenge and the activated immune status of the innate cells, T cells and B cells in obese versus lean individuals. The data fusion approach was able to detect cells that do not individually show a difference between clinical phenotypes but do play a role in combination with other cells.

## Introduction

Multicolour flow cytometry (MFC) is a powerful analytical platform used to measure multiple cellular markers in potentially very large samples (>10,000 cells) at the single-cell level. The detection of certain cells or cellular activation states with specific combinations of expressed cellular markers may enable the discrimination of an immune response; however, the number of cellular markers usable in each MFC measurement is technologically limited by the spectral overlap between the fluorescent dyes. This limitation may reduce the cellular diversity detected within a sample, as several studies have revealed that simultaneously combining more markers leads to a more detailed view of the immune system in which a greater cell variability can be discerned^1^.

Ideally, the complete cellular heterogeneity should be measured using single-cell omics^2^. Cells with specific marker expressions that are not by themselves predictive may support predictions based on other cells and the covariance between the cell types. Multivariate data analysis methods should only highlight the relevant cellular variability for the studied clinical phenotype. A single cell (sub)type may not describe the whole system, but reporting the complete cellular variability may mask the most important differences in the studied clinical phenotype. Moreover, the approach should ideally be data driven, enabling the identification of not only the cells predicted by the research hypothesis, but also the discovery of new cell (sub)types involved in the studied clinical phenotype.

To increase the number of measured parameters beyond the technical limitations of a given MFC setup, additional markers can be measured in multiple aliquots comprising different panels. Methods have been developed to integrate the non-measured marker variability in all measured aliquots on basis of their overlapping markers; however, this implies that the panels mainly characterise the same cells. Experimental research is often aimed at investigating different cell types and their activation states, such as the complementary cell types that may interact during an immunological response, which is the specific biomedical information of interest. Measuring different cell types in the same sample is often achieved using distinct panels for each blood aliquot. For this reason, we focus this article on methods that describe the cell characteristics within a panel and combine these descriptions to classify immune responses.

Existing methods for analysing multiple panels of MFC data use one of the following three approaches. The first is to cluster the cells, for example using kmeans or manual sequential bivariate gating, and to create a distribution of each sample over these clusters^3^. The second approach is to extract the important features from each marker in each panel, such as the mean, standard deviation and skewness, as is achieved using techniques such as Admire-LVQ^4^. The drawback of these two methods is that the quantitative single-cell marker expression is lost, which limits the interpretation of the data. The third approach creates 2D histograms of each pair of markers, which means the interpretation of the data is possible but tedious, as there are many pairwise combinations of markers^5^.

One of our recently developed methods, DAMACY, visualises the over- and underrepresented cells in an immune response measured in a case-control experiment^6^. This technique first creates a multidimensional histogram for each sample within a low-dimensional principal component space, which are then quantitatively compared between individuals in a second step using the discriminant analysis method Orthogonal Partial Least Squares Discriminant Analysis (OPLS-DA). OPLS-DA translates the histograms into a ‘cell map’, in which the multivariate marker expression patterns can be associated with the differential expression of the cells, which are represented as a contrast. The main advantage of this approach is that the original marker co-expressions may still be observed using this cell map, and can therefore be associated with the specific differentially abundant cells and to the diagnosis of the specific sample in which the cell is found.

The original implementation of DAMACY focussed on single-panel experiments; however, with relatively small adjustments, the second step involving the quantitative comparison of different histograms can be modified to allow the integration of data from multiple panels measured in the same samples^6^. We can extend the discriminative step of DAMACY to incorporate the fusion of data from multiple measured aliquots to discern groups of samples based on the covariance between the cell populations they express within each panel, and the relationships between the cell populations in the different panels. This may enhance the predictive ability of this approach in comparison with the analysis of a single panel because of the ‘multivariate advantage’^7^. The group of cells which are only predictive when in combination with other cell (sub)types, are thus considered essential constituents in the mechanisms that allow diagnosis, but would otherwise be overlooked in a model fitted on the cells within a single aliquot. This data fusion approach may also thereby enhance the interpretation of the diagnosis, by associating a larger variability of cells with the immune response. This method of data fusion will bring flow cytometry considerably closer to a single-cell omics approach, in which the full complement of cells may be included in a diagnosis and the associated mechanistic interpretation.

## Results

We demonstrate the fusion of multiple panels of flow cytometry data from two case studies using DAMACY. The first dataset, on the *in vivo* effect of lipopolysaccharide (LPS) on gated blood neutrophils, consists of three different measured aliquots. The second dataset, investigating the effect of obesity on the immune system, was generated from two different aliquots, in which we could define three distinct subsets: T cells, B cells and innate cells, see methods for more information.

### Lipopolysaccharide challenge

The intravenous administration of LPS (endotoxin) elicits an immune response that mimics the systemic inflammatory response syndrome in humans. Upon an LPS challenge, three neutrophil subsets can be identified in the peripheral blood, which are characterised by their different expression levels of CD16 and CD62L and their co-expression of other surface markers^8^. All three panels used therefore contained the markers CD16 and CD62L, and a set of various other markers to study their co-expression in these cells during the inflammatory response.

**Figure 1:**
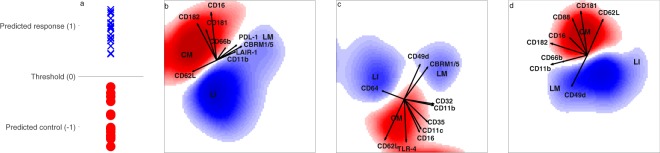
Fusion model of all the LPS aliquots after variable selection. The left panel (a) shows the LPS response samples in blue and the control samples in red. If the prediction score value is above the threshold, the samples were classified as a LPS responder. The three panels on the right (**b–d**) show the weights in the model of, respectively, aliquots 1, 2 and 3. Positive weights are coloured blue and belong to cells more represented in the LPS response samples, while negative weights are coloured red and belong to cells more abundant in the control samples. The arrows show the loadings and thus the marker expression. CM indicates mature neutrophils more abundant in the controls, while LM and LI respectively indicate mature and immature neutrophils more abundant in the LPS responders.

The individual aliquots had a decent accuracy of 98–100% for the prediction of the LPS response and control samples. The fusion model performed with 100% cross-validated accuracy, as shown in Table 1. The mean prediction score of the individual fused samples are shown in Fig. 1a, which indicates a relatively severe LPS response among all modelled samples, with higher values indicating a more severe response. The three panels on the right (b, c, and d) indicate which areas in the principal component analysis (PCA) spaces of the different measured aliquots contain cells that are predictors for LPS (blue) or the control (red). Figure 1c shows the mature neutrophils (CD62L+CD16+; **CM**) associated with the control samples, and the immature neutrophils (CD62L+CD16_dim_; **LI**) and CD62L−CD16+ neutrophils (**LM**) associated with the LPS response samples. Immature CD62L_low_ neutrophils were also observed in the first and third aliquots (Fig. 1b,d). The immature neutrophils (**LI**) exhibited a relative low expression level for most markers, but did have a relatively high level of CD64. The mature neutrophils more abundant in the control samples (**CM**) showed a higher (co-)expression of CD181, CD182, CD88 and TLR-4. The LPS-associated neutrophils (**LM**) had a higher (co-)expression of PDL-1, CBRM1/5, LAIR-1, CD32, CD49d, CD11b and CD66b. Another small subpopulation associated with the control samples could also be identified within aliquots 2 and 3, but was set to zero by the variable importance in projection (VIP) step (see Supplementary Fig. 1c,d). This subpopulation had a similar expression pattern to the LPS-associated mature neutrophils, but had a higher level of CD11b, CD11c, CD32, CD35, CD49d, CD66b and CD182 expression. This subpopulation was more abundant in the control samples in some individuals, while in other individuals the subpopulation was more abundant in the response sample, which would explain the lower prediction specificity of aliquots 2 and 3.

**Table 1:** Cross-validated performance of the models for each dataset and the fusion of all three datasets.

	Accuracy	Sensitivity	Specificity
Aliquot 1	100%	100%	99%
Aliquot 2	97%	98%	97%
Aliquot 3	97%	100%	94%
Fusion	100%	100%	99%

### Effect of obesity on the immune system

In this study, we wanted to identify the differences in the circulating immune cells of obese and lean individuals, based on markers measured in multiple aliquots. Discriminant models were separately built for each aliquot, as shown in Table 2. The models built on the innate cells and the T cells were able to discriminate obese from lean individuals (75% and 79% accuracy, respectively), while the model built using the B cell data had a lower predictive accuracy of 71%. The inclusion of the innate cell data resulted in a large increase in accuracy compared to models using only the B and T cells, while the combination of data from the B or T cells with the other groups resulted in a much smaller increase in accuracy. This suggests that the innate cells describe a unique variance predictive of obesity not present in the B and T datasets. The B cell data only made a minor contribution to the model, although in combination with data from the T cells and innate cells the B cell data did support the discrimination between obese and lean individuals.

**Table 2:** Performance of the DAMACY models on each dataset in the obese versus lean study, and the fusion of all three datasets.

Dataset	Accuracy	Sensitivity	Specificity	p-value	%increase
B cells	71%	80%	59%	<12/1000	1.5%
T cells	79%	86%	70%	<1/1000	4.5%
Innate	75%	75%	75%	<9/1000	11.8%
Data fusion	81%	82%	80%	<2/1000	

Accuracy indicates the percentage of correctly classified samples in the cross-validation study, sensitivity reflects accuracy in identifying the obese samples and specificity indicates the ability to detect control (lean) samples. The p-value is the relative amount of higher prediction accuracies found after 1000 permutations. The %increase values reflect how much the accuracy would increase if that dataset was included in the data fusion model when compared with a fusion model comprising only the two other datasets.

**Figure 2:**
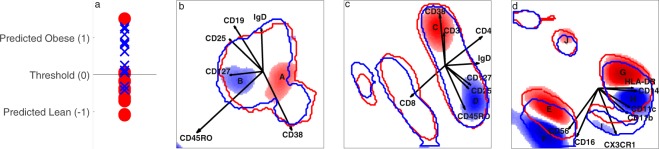
Fusion model after variable selection. The left panel (a) shows the obese individuals (blue crosses) and the lean controls (red circles). If the predicted value was above the threshold, the individuals were classified as obese. The three panels on the right (**b**–**d**) present the weights in the model of, respectively, the B cell, T cell and innate datasets. Areas coloured blue belong to cells more abundant in obese individuals, while those in red belong to cells more common in lean (control) individuals. The blue contours show where, on average, 80% of the cells of the obese individuals lie, while the red contours indicate 80% of the cells in the lean individuals.

To further evaluate the importance of the weights of the fusion model, we used VIP to select the cellular subsets most important for discriminating between lean and obese individuals (VIP > 1). Based on this quantitative criterion, we found 10 cell populations relevant for the discrimination between these groups, as shown in Fig. 2 and summarised in Table 3. The marker expressions of these cells observed in the histograms (loadings) were confirmed by gating the areas indicated in Fig. 2 and plotting the single-marker histograms (see Supplementary Methods). Obese individuals were found to possess more activated B cells (high in CD25 and CD45RO expression), activated CD4+ T cells (high in CD25, CD45RO and CD127 expression), activated natural killer cells (NKs), classical monocytes and non-classical monocytes (higher in CD11b, CD11c and CX3CR1 expression) than the lean individuals, who had more B cells and T cells high in CD38 expression, more non-activated NK cells, and more classical monocytes (lower in CD11b, CD11c and CX3CR1 expression). Lean individuals also possessed more cells with a low to absent expression of the measured markers, which may be plasmacytoid dendritic cells (pDCs; characterised by a low expression of CD11b and CD11c). As shown by the blue and red contours, some cell populations were not useful in the discrimination of obese and lean individuals, including the CD38−IgD+B cells, B cells with very high CD38 expression, CD8+ CD4− cells, (non)-activated intermediate monocytes and non-activated non-classical monocytes, in addition to the cell population which was negative for all markers.

**Table 3:** Cell populations found in Fig. 2.

Aliquot	Letter	Cell type	Marker expression	More abundant in
B cells	A	B cells	CD38+CD45RO−CD25−CD127−	Lean
B cells	B	B cells	CD38+CD45RO+CD25+CD19++CD127_dim_	Obese
T cells	C	CD4+ CD8−	CD38+CD45RO−CD25−CD28+CD3++	Lean
T cells	D	CD4+ CD8−	CD38−CD45RO+CD25+CD127+CD28++CD3+	Obese
Innate	E	NK cells	CD56+CD16+CD14−HLADR−CD11b_dim_CD11c_dim_CX3CR1_dim_	Lean
Innate	F	NK cells	CD56++CD16++CD14−HLADR−CD11b_dim+_CD11c_dim+_CX3CR1_dim+_	Obese
Innate	G	Classical monocytes	CD56−CD16−CD14+HLADR+CD11b+CD11c_dim+_CX3CR1_dim_	Lean
Innate	H	Activated classical monocytes	CD56−CD16−CD14+HLADR++CD11b++CD11c+CX3CR1+	Obese
Innate	I	Activated non-classical monocytes	CD56−CD16++CD14_dim_HLADR++CD11b_dim+_CD11c++CX3CR1++	Obese
Innate	J	plasmacytoid dendritic cells?	CD11c_dim_CD11b_dim+_, rest negative	Lean

The expression of the markers are summarised in five different categories, from lowest to highest: −, dim, dim+, +, ++. The letters correspond to the same letters as in Fig. 2.

#### Comparison with other methods

**Figure 3:**
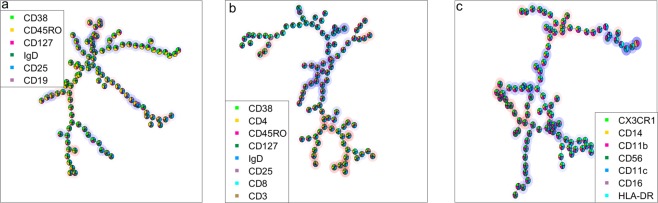
Self-organising maps of, respectively, the B cell (**a**) T cell (**b**) and innate cell (**c**) datasets. The relative marker expression of a node is depicted as a pie chart. Blue shading behind the node indicates cell populations more abundant in obese individuals, while those with red shading contain cells less abundant in obese individuals, as predicted with SVM.

Table 4 shows the performance of different methods in the discrimination between lean and obese individuals. The combination of describing the cellular distribution with self-organising maps (SOM) and support vector machines (SVM) as classifiers resulted in an equal model performance to that of the fusion performed with DAMACY; however, the interpretation of the model results is considerably more challenging for approaches using SOM and SVM. The SOM + SVM approach resulted in three sets of 100 nodes, almost all of which appeared to be important for the discrimination of lean and obese individuals, as seen in Fig. 3. The use of Citrus circumvents the interpretation issue of having too many nodes by putting a lasso regularisation on the regression coefficients, resulting in only a few nodes identified as being important for discrimination. However, in the double cross-validation, the combination of SOM + lasso regularised logistic regression did not seem to perform well in terms of accuracy, despite resulting in a significant model according to the p-value. This is probably because the number of variables (nodes) for classification is drastically reduced in this approach, meaning the heterogeneity of both classes is not captured well. Furthermore, the combination of OPLS-DA + SOM did not perform well, although OPLS-DA did perform well as part of the DAMACY algorithm. The histogram bins close to each other are highly correlated because of the smoothing step in DAMACY, and OPLS may take advantage of these correlations.

**Table 4:** Performance of the different methods on all three datasets of the lean versus obese model.

	Accuracy	Sensitivity	Specificity	p-value
Fusion with DAMACY	81%	82%	80%	<2/1000
SOM^16^ + SVM^19^	81%	66%	94%	<1/1000
DAMACY base^6^ + SVM^19^	77%	83%	71%	<1/1000
Admire-LVQ^4^	73%	75%	71%	<13/1000
SOM^16^ + lasso regularised logistic regression^19^	73%	73%	74%	<3/1000
SOM^16^ + OPLS-DA^20^	71%	66%	77%	<14/1000

Accuracy indicates the percentage of correctly classified samples in the cross-validation study, sensitivity reflects accuracy in identifying the obese samples and specificity indicates the ability to detect control (lean) samples. The p-value is the relative amount of higher prediction accuracies found after 1000 permutations.

The DAMACY base model did not perform well using SVM as a classifier, which is probably because of the overfitting caused by the many correlated variables (bins). This also prevented the lasso regularised logistic regression from converging. Admire-LVQ did not perform well, possibly because of the feature extraction of every single marker, which means the multivariate advantage does not apply. The Admire-LVQ approach could not identify activated NK cells (CD11b_dim_CD56+CD16+), but did detect a high kurtosis of CD127 in the T cells of the obese individuals. To conclude, both the fusion with DAMACY and the SOM + SVM approaches performed well in terms of prediction accuracy; however, the fusion with DAMACY was more easily interpreted, as shown in Fig. 2 and Table 3.

## Discussion

The data fusion extension of DAMACY shows an increased predictive and interpretative capability compared with the separate analyses of different measured aliquots. Although it had no increased predictive ability in the analysis of the LPS dataset, this approach did reveal that the marker CD64 in aliquot 2 resulted in a clear separation between two LPS-associated neutrophils, whereas the other two aliquots showed a continuum. The fusion guaranteed that this cellular signature is significant across all measured aliquots, but was less influenced by a non-discriminating cell subtype in aliquots 1 and 3. In the obese versus lean dataset, we observed a significant improvement in prediction using the fusion DAMACY approach, although some individuals were still misclassified. This misclassification may have occurred because those individuals are either not affected by obesity in the same way as the other individuals, or because their immune system is affected by something other than obesity. The prediction score correlated with obesity related clinical parameters such as weight, waist circumference and fat percentage, but not with other clinical parameters, see Supplementary Table 2. The B cells on their own were marginally significantly different between obese and lean people, but were found to enhance the predictive ability of the model when combined with the T cells or innate cells.

Variable Importance on Projection (VIP) provides a quantitative identifier for cells that are important for the prediction, and is calculated based on the weights of the final (O)-PLS model. Another approach is to apply sparsity at the same time as building the model, as is used in the lasso regularised logistic regression; however, this may not be sufficient to model the comprehensive biological change in the clinical phenotypes. The combination of fusing the histograms of the PCA models of the different measured aliquots with the application of VIP may help to reveal all the relevant components of the immune response that could be distinguished using the measured markers. This improved overview is provided by the generation of a map for each aliquot, in which only the relevant cells for either the control or the case are highlighted, overlaid with the original marker contribution as loadings of the PCA model.

A previous hypothesis-driven analysis of the lean versus obese dataset with sequential 2D gating in combination with univariate testing^9^ resulted in the same conclusions as those reported here using the DAMACY analysis, *i.e*., obese people had higher CD11b expression levels on their classical monocytes, higher CX3CR1 expression levels on their non-classical monocytes and relatively more NK cells. Moreover, the hypothesis-driven analysis also identified a higher CD11b expression level on the intermediate monocytes, which were not found to be discriminative in the DAMACY analysis because they were not highlighted after the VIP correction; therefore, the full dataset did not contain sufficient power to indicate these cells are significant contributors to prediction. The hypothesis-driven analysis failed to find any significant changes in the B and T cells, possibly because these cell populations were not significantly different between lean and obese individuals on their own; for example, the CD25+CD45RO+ B cells had a p-value of 0.0555 between the groups, but were predictive in combination with other cells. The classification of the fusion model does improve (by 1.5%) when the B cell data were included, and the resulting model contained weights for this dataset. Supplementary Fig. 9 shows how these cells (**B** in Fig. 2) may help to separate the lean and obese individuals when combined with the non-classical monocytes (**I** in Fig. 2). Another reason could be that the specific subset was not tested in the hypothesis-driven analysis, as was the case for the increased abundance of CD25− CD45RO− B cells (p-value 0.0024) and CD4+CD38− CD45RO+CD25+CD127+ T cells (p-value 8.3e-4) and the decreased amount of CD4+CD38+CD45RO− CD25−CD127− T cells (p-value 0.0011) in the obese individuals. For this reason, a data-driven approach is better suited to the study of currently unknown interactions between cells, as it will detect all cell types with significant differences in abundance between the studied clinical phenotypes, whereas hypothesis-driven research will only identify differences in the cells that are tested.

Multivariate analysis methods observe the expression of all markers on all cells for every patient. The importance of the cells within the model is then determined by (1) their marker expression in relation to the investigated immune response or (2) their co-occurrence with other cells. Standard multivariate methods, including SVM, SOM, PCA, and PLS, provide a readout of the relative importance of every cell in the model, which leads to a data-dense representation (see *e.g*., Fig. 3) from which the dedicated immunological information may be challenging to interpret. Sparse methods, such as the lasso regularised logistic regression used in Citrus, have been developed to specifically provide descriptive models for an immune response involving as few cell types as possible. The resulting minimal set of cells is much easier to interpret than results from the standard methods, but do not contain the comprehensive set of cells involved in the response. Immune responses may consist of highly specific mechanisms, but may also involve generic mechanisms such as normal inflammatory markers that may be induced for many different immune responses; the involvement of both aspects in the immune response are therefore redundant. Sparse models do not distinguish between the specificity of such patterns in a binary comparison, such that strongly responding but highly generic cells may be identified as the most characteristic for a studied response. Second, and most characteristic in our study, is that the sparsity criterion may select different redundant cells for different realisations within the double cross-validation.

The optimal cell set from a comprehensive cell characterisation would include only the relevant comprehensive subset for the response. DAMACY, specifically involving OPLS as the top model, is among the most appropriate of the described methods for this aim. The method is data-driven, and thus may also identify unexplored relationships between (sub)types of cells and the studied response. OPLS removes the orthogonal information and, together with VIP, only shows the relevant cellular variability. Finally, complementary cell data may be merged in a multiblock sense, and may also be extended to clinical or other omics data. The set of cells identified using DAMACY may then be considered to represent all cells that are significantly involved. This may be compared to the objective of metabolomics, which (1) aims to measure all small organic metabolites and (2) tries to identify all chemical species involved in the studied biological process. Analogously, the cell set resulting from the DAMACY analysis will be optimally informative for further immunological studies on the variability of, and mechanisms underlying, the studied response.

## Conclusion

The data fusion extension of the DAMACY method can be used to effectively discriminate clinical phenotypes from flow cytometry data with multiple aliquot measurements using different panels. The method uses correlations between cell subtypes and generates a cell map for each aliquot with only the relevant cells highlighted for discriminating between the clinical phenotypes. The original (co-)expression of the marker is overlaid as a vector on the map, enabling researchers to clearly observe which cells are important for the clinical phenotypes and their (co-)expression. This data fusion method performs equally well or better than the best DAMACY model using a single aliquot, as statistically tested with a double cross-validation and permutation testing.

## Methods

### Data

Peripheral blood was extracted from subjects in both the LPS challenge study and obese versus lean study, all of whom gave their written informed consent before participating. All data were obtained using standardised protocols. The LPS challenge study and sample collection were approved by the medical ethics committee of Radboud University Medical Center (Radboudumc) Nijmegen, The Netherlands. The study protocol of the obese versus lean study was approved by the Medical Ethical Committee Jessa hospital, Hasselt, and Hasselt University, Belgium. Both studies were performed in accordance with the Declaration of Helsinki (Forteleza, 2013).

#### LPS data

In total, blood samples were taken from 16 individuals before and after 180 minutes of LPS administration. Three aliquots containing the following markers were measured:

Aliquot 1: CD182, CD181, CD62L, CD66b, PDL-1, LAIR-1, CBRM1/5, CD11b, CD54, CD16;

Aliquot 2: CD35, TLR-4, CD62L, CD11c, CD49d, CD32, CBRM1/5, CD11b, CD64, CD16;

Aliquot 3: CD88, CD181, CD62L, CD66b, CD49d, CD182, CD11b, CD16.

The data were gated for neutrophils using forward, sideward scatter, and CD16 expression using a standard protocol.

#### Obesity versus lean data

In total, blood was extracted from 31 individuals, of whom 13 were lean with a body mass index (BMI) of between 20.83 and 25.62, and 16 were obese with a BMI between 30.47 and 49.27^9^. Two aliquots containing the following markers were measured:

Aliquot 1: CD38, CD4, CD45RO, CD127, CD28/IgD, CD25, CD8/CD19, CD3;

Aliquot 2: CXRCR1, CD14, CD56, CD11c, CD16, HLA-DR, CD3/CD19/CD66b.

Aliquot 1 contains specific antibodies for B cells and T cells on the same fluorophore, and was split to simplify the interpretation of the data by gating for B cells (CD19+CD3−) and T cells (CD3+). The gated data contained the following markers:

B cells: CD38, CD45RO, CD127, IgD, CD25, CD19;

T cells: CD38, CD4, CD45RO, CD127, CD28, CD25, CD8, CD3.

Aliquot 2 was gated for innate cells by removing all cells positive for the markers CD3, CD19 and CD66b.

#### Experimental method

In this paper, the fusion of the histograms in DAMACY are compared with the prediction with extracted features in Admire-LVQ^4^, and with the approaches in which the data are first clustered using SOMs and subsequently classified using OPLS-DA, SVM or a lasso regularised logistic regression. SOM was selected because it has been proven to be a very effective clustering method, both in terms of describing the data and speed^10,11^, which is required to effectively cross-validate the algorithms. SVM successfully classified data in the Flowcap 2 challenge^5^, while the lasso regularised logistic regression successfully classified data in Citrus^12^. Both the prediction performance and the interpretation of the visualized data were compared.

#### Cross-validation and permutation

The data were split into training and test sets using a five-fold cross-validation across 50 iterations. The data were thus randomly stratified into five parts, four of which were used to train each model, while the fifth was used to assess the model performance. This was repeated such that all parts were used once to assess the model performance. The stratified random splitting of the data into five parts was repeated 50 times^13^. The composition of the training and test sets were stored and reused to assess the performance of each method, resulting in a fairer comparison between the methods. OPLS involves an internal leave-one-out cross-validation to determine the number of orthogonal latent variables, while the lasso regularised logistic regression had an internal ten-fold cross-validation to determine lambda^14^.

Permutation testing was applied to determine whether the built models predicted an actual effect or random effect. The labels were randomly permuted 1000 times and models were built to classify the random effects. In OPLS, the same number of orthogonal latent variables were used as determined in the model, while in lasso the same lambda was used, so that an internal cross-validation loop was not required. A p-value can be calculated by counting the number of times the accuracy of a random effect is equal to or higher than the accuracy of the studied effect and dividing by the total number of permutations, in this case 1000 (see Eq. 1)^14^.

1 $$p < \frac{\sum (accurac{y}_{permutated}\ge accurac{y}_{model})+1}{1000}$$

#### Pre-processing of the data

The data of each aliquot measurement were transformed using the hyperbolic inverse sine with a cofactor of 150. In the case of the obese versus lean study, the data were transformed with median centring using the median marker expression of all lean individuals from the training set, and subsequently scaled by dividing every marker by its standard deviation in the lean individuals from the training set. In the case of the LPS dataset, each measurement was centred and scaled using the median and standard deviation of each individual from the measurements both before and after LPS.

#### Adaption of DAMACY

After pre-processing, a PCA model was built on the concatenated training data, and all individuals were projected into the principal component space. A 2D histogram was calculated on the PCA scores of each aliquot, as described in the original DAMACY paper^6^ and summarised in the supplementary material of this manuscript. The histograms of each aliquot were smoothed and normalised to the unit sum of all bins, after which the unfolded histograms of each aliquot were concatenated into one large matrix (see Eq. 2).

2 $${\bf{H}}=[\begin{array}{ccc}{{\bf{H}}}_{1,1,1} & \cdots \,{{\bf{H}}}_{1,{F}^{2},1}\,\cdots  & {{\bf{H}}}_{1,{F}^{2},M}\\ \vdots  & \ddots \,\vdots \,\ddots  & \vdots \\ {{\bf{H}}}_{I,1,1} & \cdots \,{{\bf{H}}}_{I,{F}^{2},1}\,\cdots  & {{\bf{H}}}_{I,{F}^{2},M}\end{array}]$$

where **H**_*ifm*_ is a bin (*f*) in the unfolded histogram (*m*) of individual $i=1\ldots I$, *I* is the total number of individuals, $f=1\ldots {F}^{2}$, *F* is the total number of bins in one dimension of the histogram of aliquot $m=1\ldots M$ and *M* is the total number of different measured aliquots. The default *F* value used is *F* = 500, but in this article 100 bins were used, because the number of cells per sample was relatively low (<10,000).

The large matrix **H** was mean-centred using the mean of the training set. An OPLS-DA model was built using the training data of **H** and the logical dummy vector with length I*, where I* is the number of individuals in the training data. A final model was then built on all samples, giving the weight vector ***w***_*top*_, which can be refolded into an $(F\times F\times M)$ matrix **W**_**top**_ when the number of principal components is ${{\boldsymbol{K}}}_{{\bf{b}}{\bf{a}}{\bf{s}}{\bf{e}}}=2$ in the base model. After building the OPLS-DA model, the VIP^15^ was calculated for each bin weight, using Eq. 3. Weights (histogram bins) with a VIP lower than one were set to zero, while weights with a VIP greater than one were the histogram bins that contributed significantly to the model.

3 $${\rm{VIP}}=\sqrt{{F}^{\ast }\times {{\boldsymbol{w}}}_{top}^{2}}$$

The matrix **W**_**top**_ corresponds to the original *M* histograms ${{\bf{H}}}_{{{\boldsymbol{i}}}_{{\boldsymbol{m}}}}$, forming a leukocyte map for each aliquot (see Figs 1 and 2). This map shows which histogram bins, hence which cells, are over- or underrepresented in either group, and can therefore be interpreted together with the mean predictive scores of $\,\hat{{\boldsymbol{y}}}$. Each single cell positively or negatively alters the individual score when it ends up in a positively or negatively weighted bin in the leukocyte map. Finally, a prediction score $\hat{{\boldsymbol{y}}}\,\,$was calculated for each individual in the test set. The whole algorithm was repeated until every iteration of the training set matrix was used. This resulted in 50 values of $\hat{{\boldsymbol{y}}}$ for each individual. The mean value was plotted as the mean prediction score.

#### Self-organising maps

The SOMs were built on the training data of each aliquot using the Matlab toolbox for SOMs^16^, with the same default setting as flowSOM^10^, including the Euclidian distance, a grid size of 10 by 10 nodes, and the batch training algorithm with a number of steps 10 times greater than the number of cells in the training set. Finally, the number of cells per node was calculated for all samples using the nearest neighbour, resulting in a large matrix with a size of *I* × 100 *M*, where *I* is the total number of individuals and *M* is the number of aliquots.

#### Classifiers on self-organising map nodes

Three classifiers were tested on the large matrix; SVMs, as used in flowpeakSVM^3,5^, the lasso regularised logistic regression used in Citrus^12^ and the OPLS-DA in DAMACY^6^. For the SVM, a linear kernel and a box constraint of one were used. The regression coefficients of SVM, the regression coefficients with lambda and the minimum deviance in the inner cross-validation loop of the lasso regularised logistic regression, and the weights of the OPLS-DA model with the number of orthogonal variables that gave the minimum error in the inner cross-validation loop were used for visualization. A minimum spanning tree was built for each SOM using the kamada and kawai algorithms^17^, as used in flowSOM^10^ and SPADE^18^. Each node was coloured according to its regression coefficient or weight and the mean marker expression as a pie chart.

## Supplementary information

Supplementary info

## Data Availability

The datasets generated and/or analysed during the current study are available from the corresponding author upon reasonable request.
