# Supplementary material for: A novel data fusion method for the effective analysis of multiple panels of flow cytometry data
Source: Sci Rep. 2019 May 1;9:6777. doi: 10.1038/s41598-019-43166-x (PMC6494873; doi:10.1038/s41598-019-43166-x)
Supplement: Supplementary file 1 — Supplementary info [file 41598_2019_43166_MOESM1_ESM.docx]

**A novel data fusion method for the effective analysis of multiple panels of flow cytometry data***Gerjen H. Tinnevelt^1,2^, Selma van Staveren^2,3^, Kristiaan Wouters^4^,* Erwin Wijnands^5^,  *Kenneth Verboven^6,7^, Rita Folcarelli^1^, Leo Koenderman^3^, Lutgarde M.C. Buydens^1^, Jeroen J. Jansen^1^*

*^1^Radboud University, Institute for Molecules and Materials (Analytical Chemistry), postvak 61 P.O. Box 9010, 6500 GL Nijmegen, The Netherlands*

*^2^TI-COAST, Science Park 904, 1098 XH Amsterdam, The Netherlands*

*^3^Department of Respiratory Medicine and laboratory of translational immunology (LTI), University Medical Center Utrecht, Heidelberglaan 100, 3584CX, Utrecht, The Netherlands*

*^4^Deptartment of Internal Medicine Laboratory of Metabolism and Vascular Medicine, P.O. Box 616 (UNS50/14), 6200 MD Maastricht, The Netherlands*

*^5^Experimental Vascular Pathology group, P.O. Box 5800, 6202 MZ Maastricht, The Netherlands*

*^6^REVAL - Rehabilitation Research Center, Faculty of Rehabilitation Sciences, Hasselt University, Diepenbeek, Belgium*

*^7^BIOMED - Biomedical Research Institute, Faculty of Medicine and Life Sciences, Hasselt University, Diepenbeek, Belgium.*


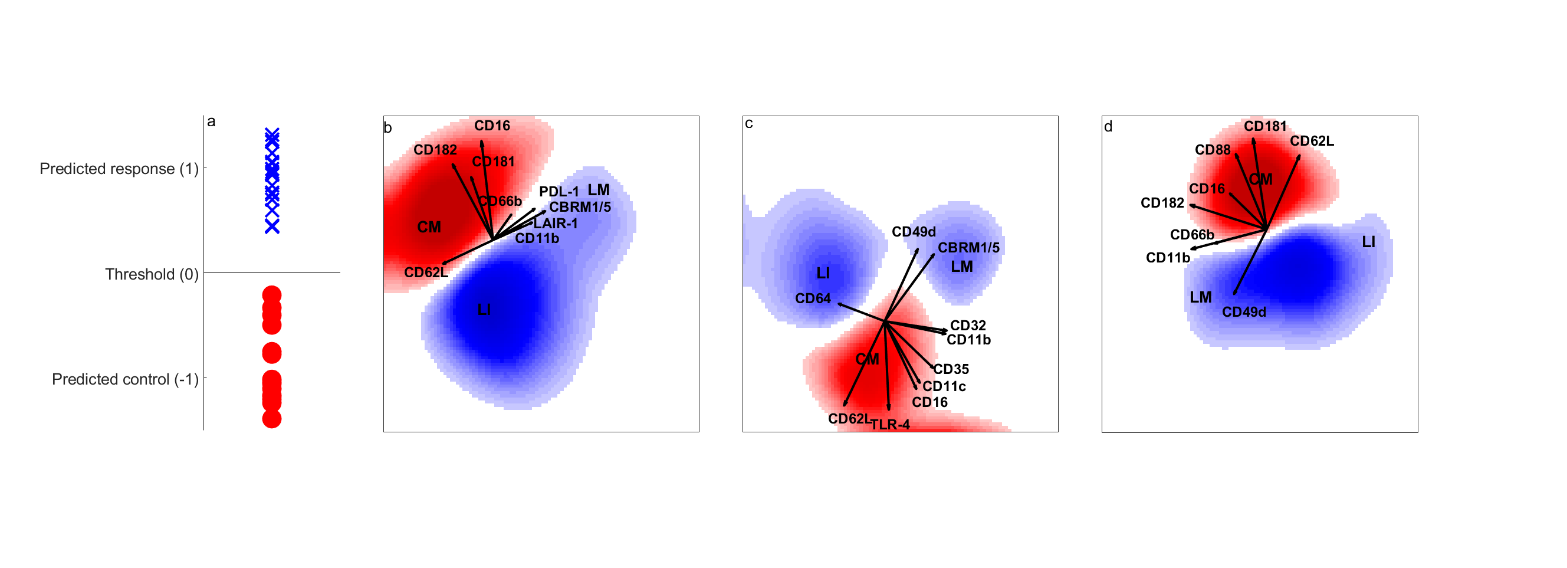


Figure 1: Fusion model of all the LPS aliquots after variable selection. The left panel (a) shows the LPS response samples in blue and the control samples in red. If the prediction score value is above the threshold, the samples were classified as a LPS responder. The three panels on the right (b, c, d) show the weights in the model of, respectively, aliquots 1, 2 and 3. Positive weights are coloured blue and belong to cells more represented in the LPS response samples, while negative weights are coloured red and belong to cells more abundant in the control samples. The arrows show the loadings and thus the marker expression. CM indicates mature neutrophils more abundant in the controls, while LM and LI respectively indicate mature and immature neutrophils more abundant in the LPS responders.


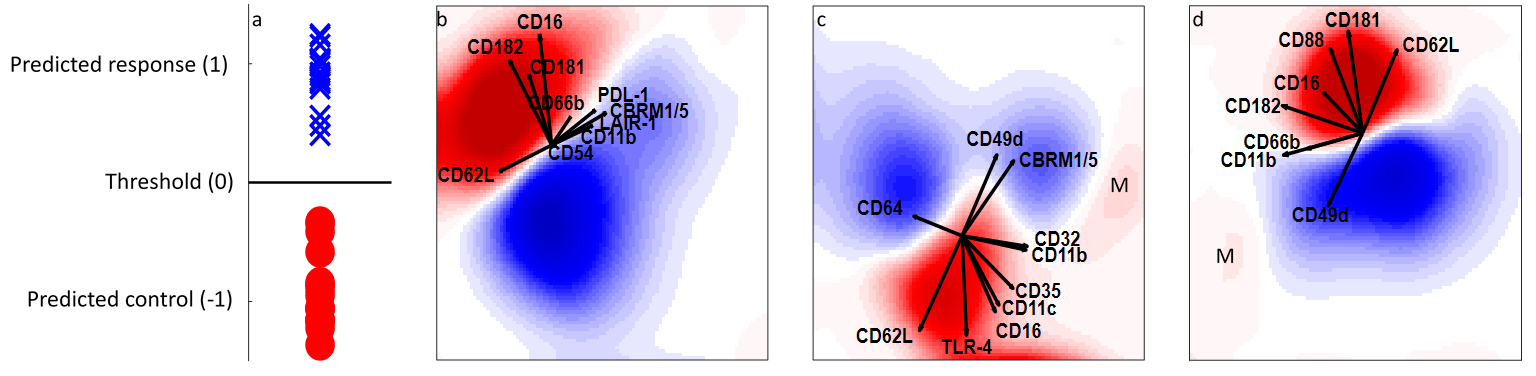


Supplementary Figure 1: Fusion model of all the LPS aliquots. The left panel (a) shows the LPS response samples in blue and the control samples in red. If the prediction score value is above the threshold, the samples were classified as a LPS responder. The three panels on the right (b, c, d) show the weights in the model of, respectively, aliquots 1, 2 and 3. Positive weights are coloured blue and belong to cells more represented in the LPS response samples, while negative weights are coloured red and belong to cells more abundant in the control samples. The arrows show the loadings and thus the marker expression. The small cell population indicated by M (panel c and d) was removed by variable importance in projection (VIP) in Figure 1.

Supplementary Table 1: Performance of the different methods on all three aliquots of the LPS dataset

|  | Accuracy | Sensitivity | Specificity |
| --- | --- | --- | --- |
| Fusion with DAMACY | 99% | 100% | 99% |
| SOM[^15^](#_ENREF_15) + SVM[^18^](#_ENREF_18) | 99% | 100% | 99% |
| DAMACY base[^6^](#_ENREF_6) + SVM[^18^](#_ENREF_18) | 98% | 98% | 99% |
| SOM [^15^](#_ENREF_15)+ Lasso regularized logistic regression[^18^](#_ENREF_18) | 93% | 92% | 95% |
| SOM[^15^](#_ENREF_15) + OPLS-DA[^19^](#_ENREF_19) | 100% | 100% | 100% |


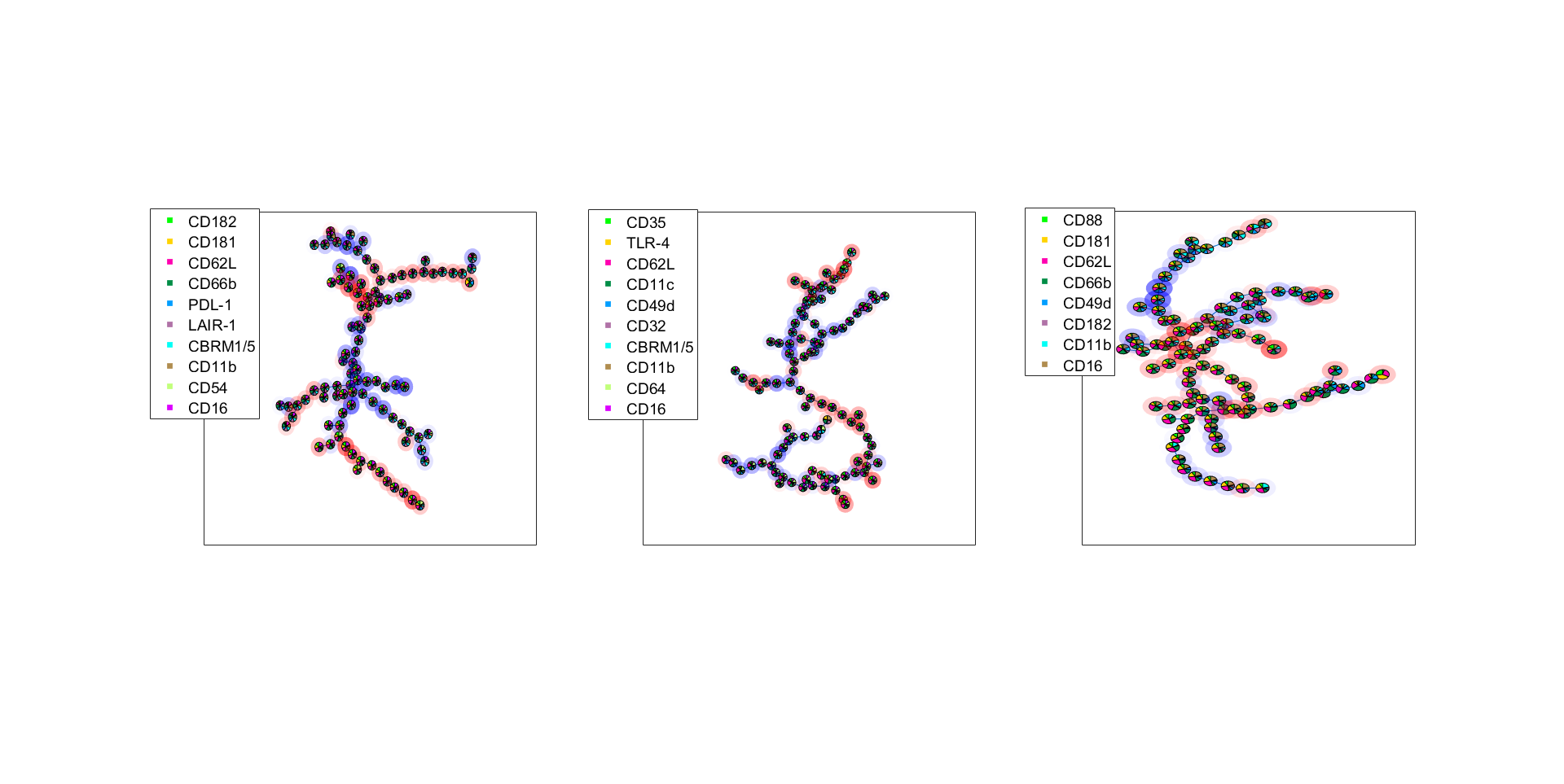


Supplementary Figure 2: Self-organizing map of the LPS dataset. The relative marker expression of a node is depicted as a pie chart. Blue shade behind the node contain more cells in obese individuals and the red shade contain less as predicted with SVM.


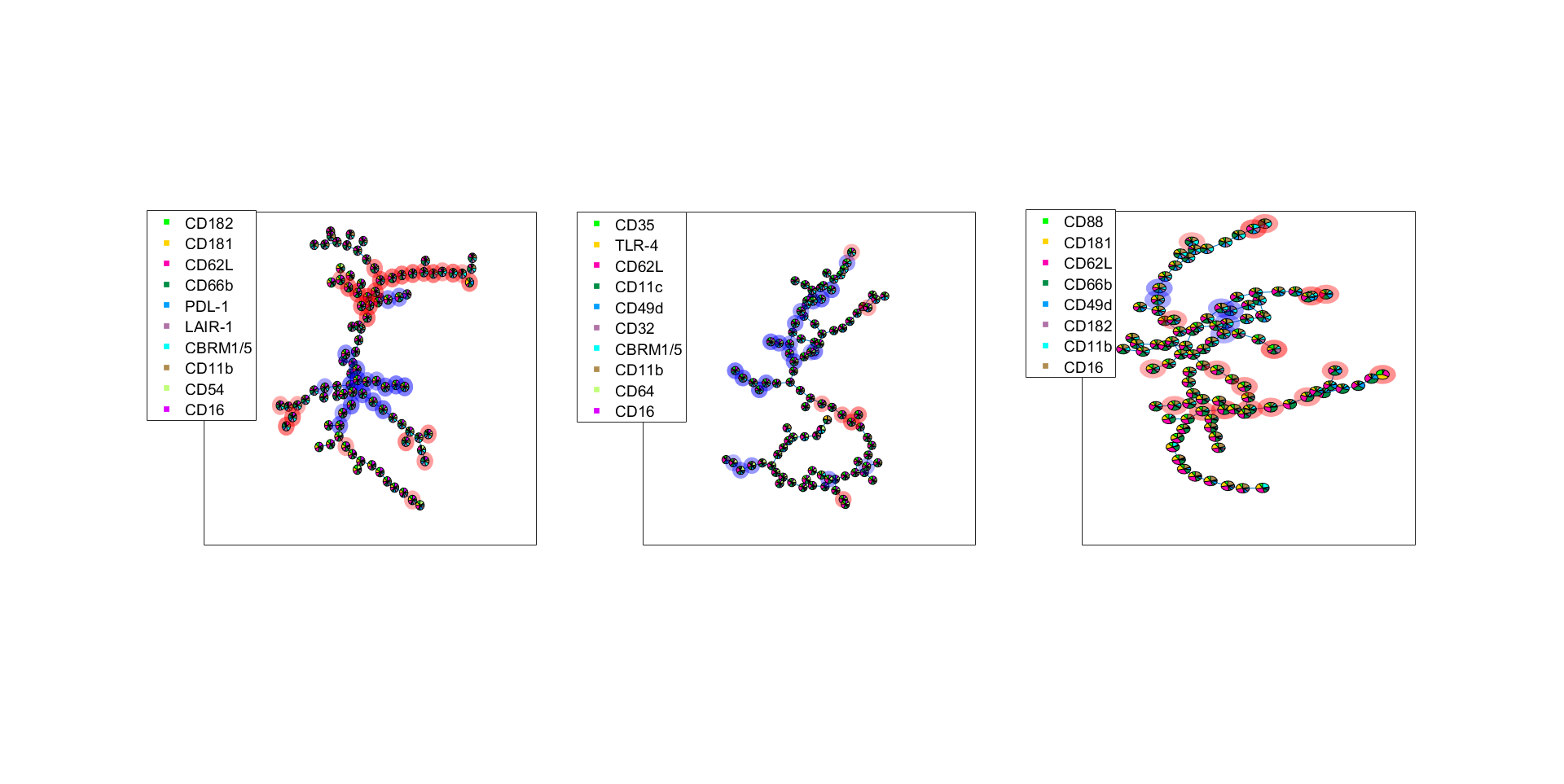


Supplementary Figure 3: Self-organizing map of the LPS dataset. The relative marker expression of a node is depicted as a pie chart. Blue shade behind the node contain more cells in obese individuals and the red shade contain less as predicted with OPLS.


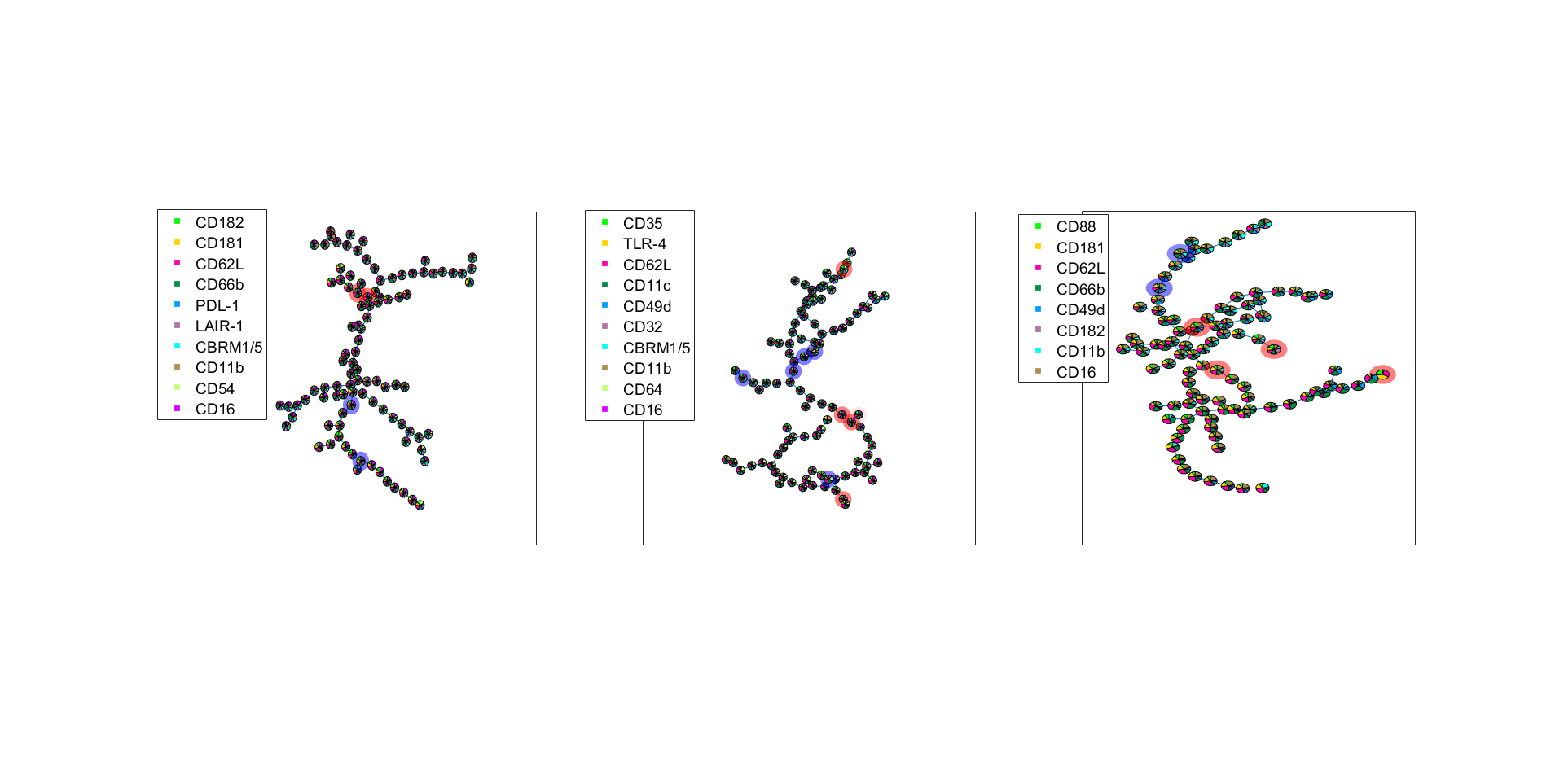


Supplementary Figure 4: Self-organizing map of the LPS dataset. The relative marker expression of a node is depicted as a pie chart. Blue shade behind the node contain more cells in obese individuals and the red shade contain less as predicted with Lasso.

Supplementary Figure 5: DAMACY base model with SVM as classifier. The three panels are the weights of the model of respectively the aliquot measurements of the LPS dataset. Areas colored blue belong to cells more present in LPS responders and areas colored contain cells less present in LPS responders.

Figure 2: Fusion model after variable selection. The left panel shows with blue crosses the obese individuals and with red circles the controls. If the predicted value is above the threshold, the individuals are classified as obese. The three right panels are the weights of the model of respectively the B cell, T cell and monocyte dataset. Areas colored blue belong to cells more present in obese and areas colored red belong to cells more present in lean individuals. The blue contours show where on average 80% of the cells of the obese individuals lie and the red contours of the lean individuals.


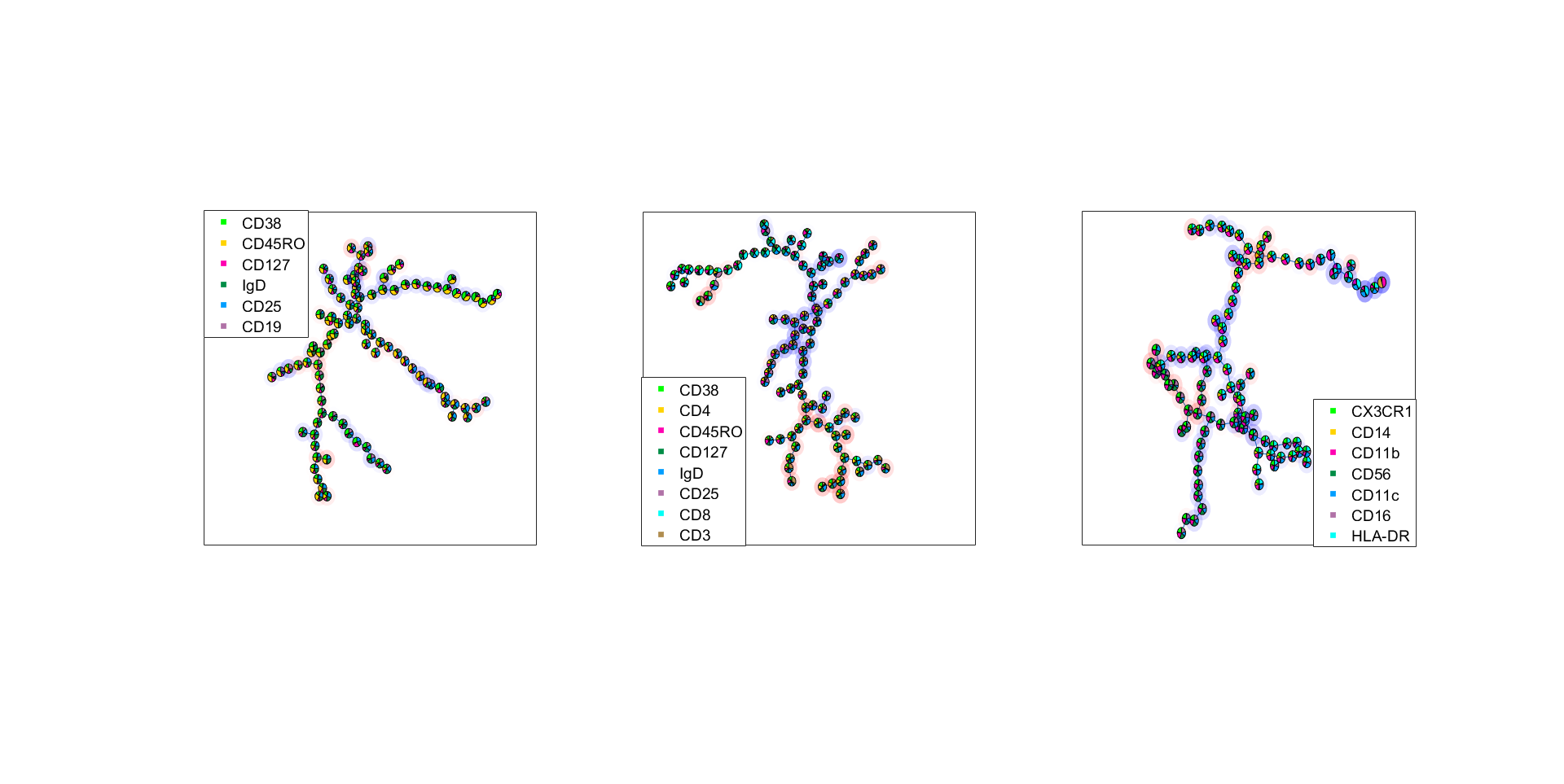


Figure 3: Self-organizing map of respectively dataset B cells, T cells and innate cells. The relative marker expression of a node is depicted as a pie chart. Blue shade behind the node contain more cells in obese individuals and the red shade contain less as predicted with SVM.


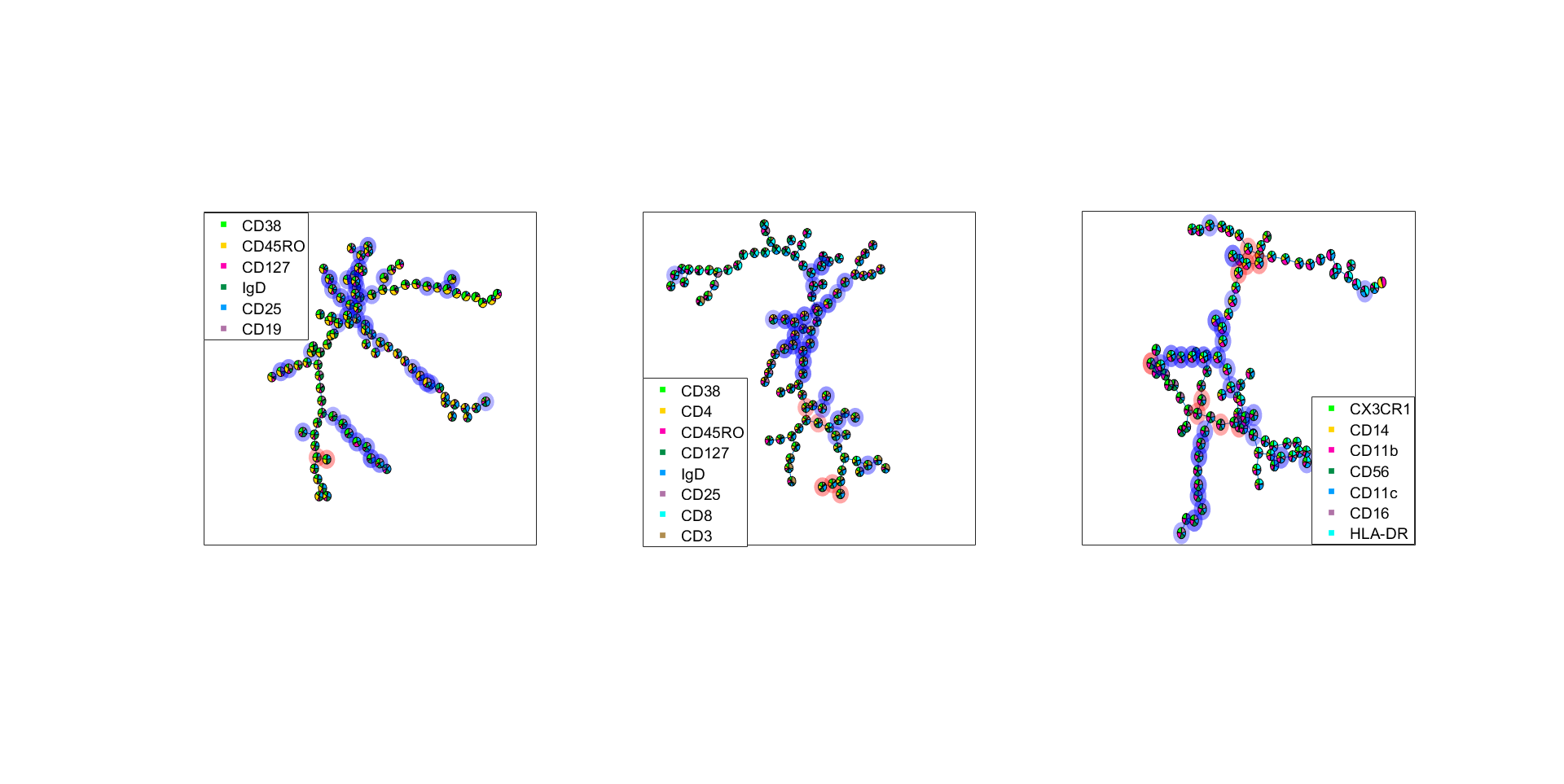


Supplementary Figure 6: Self-organizing map of respectively dataset B cells, T cells and innate cells. The relative marker expression of a node is depicted as a pie chart. Blue shade behind the node contain more cells in obese individuals and the red shade contain less as predicted with OPLS-DA.


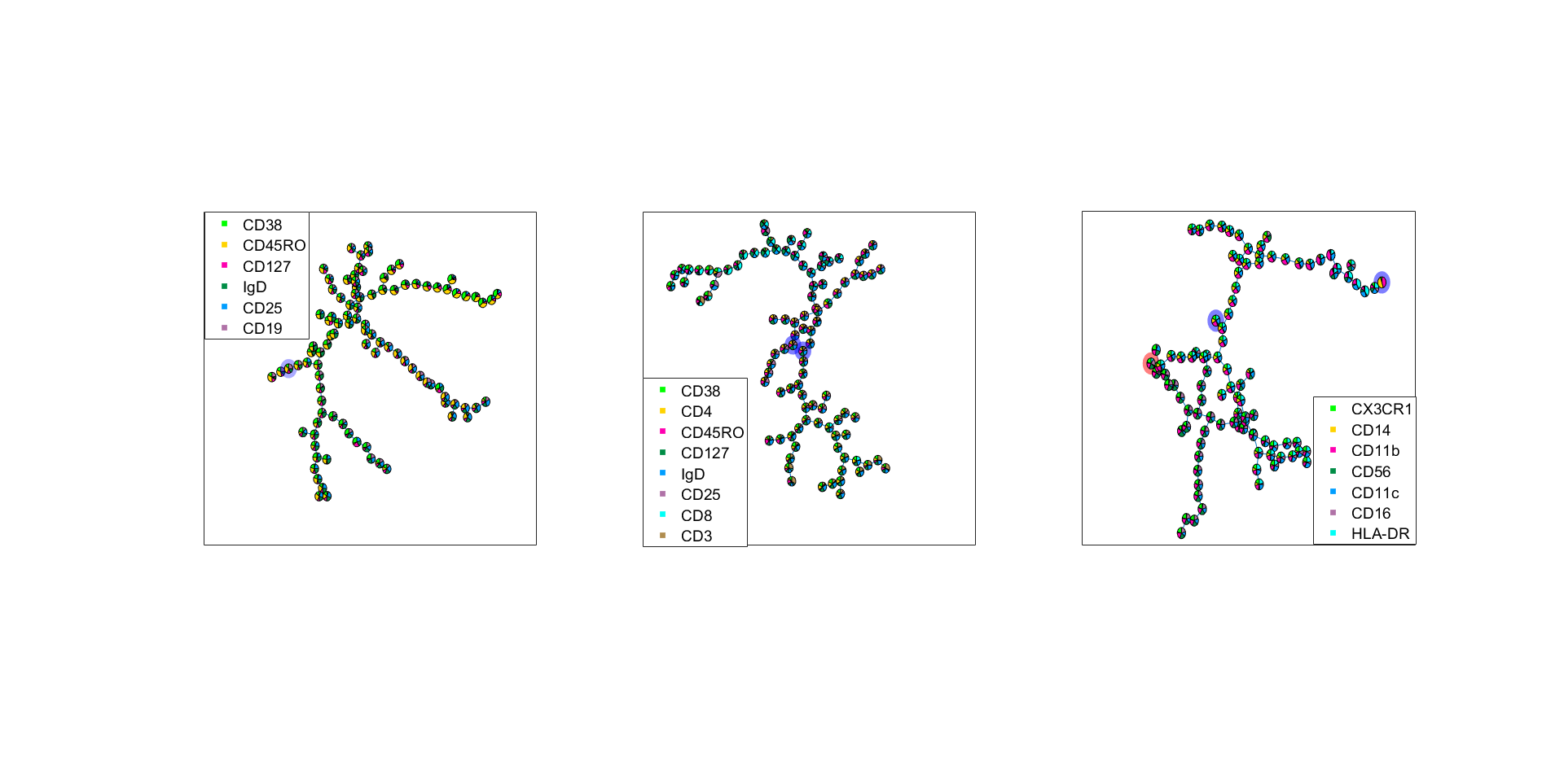


Supplementary Figure 7: Self-organizing map of respectively dataset B cells, T cells and innate cells. The relative marker expression of a node is depicted as a pie chart. Blue shade behind the node contain more cells in obese individuals and the red shade contain less as predicted with lasso regularized logistic regression.

Supplementary Figure 8: DAMACY base model with SVM as classifier. The three panels are the weights of the model of respectively the aliquot measurements of the B cell, T cell and monocyte dataset. Areas colored blue belong to cells more present in obese individuals and areas colored contain less cells.

*Supplementary Figure 9: Percentage activated B cells (Gate B in Figure 2) against the percentage non-classical monocytes (Gate I in Figure 2). The percentages are respectively based on the total B cells (positive for CD19) and total monocytes/NK cells (CD3, CD66b and CD19 negative). The red rounds represent lean individuals and the blue crosses represent obese individuals. No separation is present based on activated B cells only (x-axis). A good separation is present between non-classical monocytes (y-axis), but four obese samples are within the lean group. However, when activated B cells and non-classical monocytes are plotted against each other, the two encircled blue crosses (obese individuals) do separate from the lean group. This is probably due to negative correlation (r −0.48, p-value 0.096) in lean individuals between these two cell population, which is not present in obese individuals.*

Supplementary Table 2: Correlation between clinical parameters and the prediction score seen in Figure 2 or Body Mass Index. The correlation coefficient is given and tested on significance. Every clinical parameter above the bolded line is significantly correlated with the prediction score and with the Body Mass Index. Clearly only obesity related parameters are significantly correlated such as Body Mass Index, fat mass, waist circumference, weight, hip circumference, fat percentage, lean mass and WHR.

| Clinical parameter | Correlation with prediction score | | Correlation with Body Mass Index | |
| --- | --- | --- | --- | --- |
|  | r | p-value | r | p-value |
| Body Mass Index (kg/m²) | 0.65 | 1.3E-04 | 1.00 | 0 |
| Fat mass (kg) | 0.65 | 1.6E-04 | 0.97 | 1.5E-18 |
| Waist circumference (cm) | 0.69 | 4.0E-05 | 0.97 | 1.8E-18 |
| Weight (kg) | 0.66 | 8.9E-05 | 0.96 | 1.6E-16 |
| Hip circumference (cm) | 0.72 | 2.4E-05 | 0.94 | 1.8E-13 |
| Fat percentage (%) | 0.61 | 5.0E-04 | 0.92 | 1.9E-12 |
| Lean mass (kg) | 0.64 | 2.1E-04 | 0.85 | 7.7E-09 |
| WHR | 0.63 | 4.8E-04 | 0.84 | 3.2E-08 |
| Serum Insulin (mU/L) | 0.44 | 0.02 | 0.56 | 1.8E-03 |
| HOMA-IR | 0.38 | 0.05 | 0.53 | 4.0E-03 |
| HbA1C (mmol/mol) | 0.41 | 0.03 | 0.51 | 0.01 |
| HbA1C (%) | 0.40 | 0.04 | 0.51 | 0.01 |
| BASO (x10*9/L) | -0.40 | 0.04 | -0.43 | 0.02 |
| RETI (x10*9/L) | 0.24 | 0.23 | 0.39 | 0.05 |
| BASO (%- | -0.36 | 0.07 | -0.37 | 0.06 |
| RETI (% RBC) | 0.21 | 0.30 | 0.35 | 0.07 |
| Plasma glucose (mM) | 0.32 | 0.10 | 0.31 | 0.11 |
| Blood pressure,  Systolic (mmHg) | 0.29 | 0.15 | 0.32 | 0.11 |
| RBC (x10*12/L) | 0.24 | 0.23 | 0.31 | 0.12 |
| Blood platelets (x10*9/L) | -0.41 | 0.03 | -0.31 | 0.12 |
| WBC (x10*9/L) | -0.25 | 0.22 | -0.29 | 0.14 |
| Age (years) | -0.08 | 0.70 | -0.28 | 0.14 |
| MONO (%) | 0.16 | 0.44 | 0.28 | 0.16 |
| Hemoglobin (g/dL) | 0.23 | 0.24 | 0.25 | 0.21 |
| NEUT.SEGM (x10*9/L) | -0.28 | 0.15 | -0.25 | 0.22 |
| LUC (%) | 0.29 | 0.14 | 0.24 | 0.23 |
| EOS (%) | -0.03 | 0.86 | -0.23 | 0.24 |
| LYMF (x10*9/L) | 0.04 | 0.84 | -0.18 | 0.37 |
| BSE (mm/uur) | 0.34 | 0.08 | 0.18 | 0.38 |
| Length (m) | 0.07 | 0.72 | -0.16 | 0.39 |
| MONO (x10*9/L) | -0.16 | 0.43 | -0.12 | 0.55 |
| Blood pressure,  Diastolic (mmHg) | 0.20 | 0.32 | 0.12 | 0.58 |
| LUC (x10*9/L) | 0.12 | 0.54 | 0.03 | 0.90 |
| NEUT.SEGM (%) | -0.17 | 0.40 | -0.02 | 0.92 |
| EOS (x10*9/L) | 0.15 | 0.45 | 0.01 | 0.95 |
| LYMF (%) | 0.17 | 0.41 | 0.01 | 0.96 |

# Algorithm

Below you find the complete algorithm of Discriminant Analysis of Multi Aspect flow Cytometry data (DAMACY):

1. Import the data and arrange it as matrix $\mathbf{X}$ $\boldsymbol{=}\left[ \begin{matrix} \mathbf{X}_{1_{1}} \\ \vdots\\ \mathbf{X}_{I_{G}} \end{matrix} \right]$ of size$\left( \sum_{i_{g}=1_{1}}^{I_{G}} N_{i_{G}}\times J \right)$ for analysis by DAMACY, where $N_{i_{g}}$ is the number of cells per individual $i_{g}=1_{1},\ldots I_{1},\ldots1_{G},\ldots I_{G}$; $g=1,\ldots..G$ indicates the pre-defined groups of individuals with $g=1$ representing the control group and$g=2$ the patient/challenged group, with $j=1,\ldots,J$indicates the surface markers; The data of each individual can analogously be denoted as $\mathbf{X}_{i_{g}}=\left[ \begin{matrix} \mathbf{x}_{{1_{i}}_{g}}^{T} \\ \vdots\\ \mathbf{x}_{{N_{i}}_{g}}^{T} \end{matrix} \right]$of size $\left( N_{i_{g}}\times J \right)$ and the data of each class as $\mathbf{X}_{g}\boldsymbol{=}\left[ \begin{matrix} \mathbf{X}_{1_{g}} \\ \vdots\\ \mathbf{X}_{I_{g}} \end{matrix} \right]$of size $\left( \sum_{i_{g}=1_{g}}^{I_{g}} N_{i_{g}}\times J \right)$.
2. Use arcsinh with cofactor 150 to transform the data: $\mathbf{X}_{\log}\boldsymbol{=}arcsinh \left( \mathbf{X}/150 \right)$
3. Separate the data into test and training set using 5-fold stratified cross validation with 50 repetitions.
4. Separate the training data in a training set and a calibration set using leave one of each group out validation for small datasets or use 7 cross-validation for larger datasets (>50 individuals per group).
5. Mean centre the data using supplementary equation s1, s2 or s3. Note: only use training data to calculate mean of multiple individuals.
6. Scale the data using supplementary equation s4, s5 or s6. Note: only use training data to calculate standard deviation of multiple individuals.
7. Perform simultaneous component analysis (SCA) on training data using supplementary equation s7.
8. Project the test data onto the SCA model using supplementary equation s7C.
9. Define binsize using supplementary equation s8.
10. Create histograms $\mathbf{H}$ of size $\left( \sum_{g=1}^{G} I_{g} \times\prod_{k_{base}=1}^{K_{base}} F \right),$with $k_{base}=1, \ldots, K_{base}$ indicating the components used in the base model, $f=1,\ldots,F$ indicating the bins within the histograms, by counting the number of cells per bin per individual and normalize it using supplementary equation s9.
11. Smooth the histogram using supplementary equation s10, with $\mathbf{I}$ being the identity matrix, for matrix $\mathbf{D}_{1}$ holds that $\mathbf{D}_{1}\mathbf{H}=\Delta\mathbf{H}$and for matrix $\mathbf{D}_{2}$ holds that $\mathbf{D}_{2}\mathbf{H}=\Delta^{2}\mathbf{H}$, $\lambda$is the smoothing factor, this equation results in smoothed histogram $\mathbf{Ĥ}_{\boldsymbol{i}}$ of size $\left( \prod_{k_{base}=1}^{K_{base}} F \right)$
12. Repeat step 1-11 with all other measured aliquots and refold the smoothed histograms $\mathbf{Ĥ}$ into matrix $\mathbf{H}$ with dimensions$\left( I \times F^{K_{\mathrm{base}}}M \right)$, which results in equation 2.
13. Remove the columns (bins) in matrix $\mathbf{H}$ with small variance ($<{10}^{-6}$), resulting in matrix $\mathbf{H}^{\mathbf{*}}$ with dimensions$\left( I \times F^{*} \right)$. with $F^{*}$ being the bins with sufficient variance.
14. Perform Orthogonal Partial Least Squares – Discriminant Analysis (OPLS-DA) on train data with supplementary equation s11
15. Calculate the VIP using equation 3 and set all $\mathbf{w}_{\mathrm{top}}$ with VIP < 1 to 0.
16. Predict the test data with$ŷ\boldsymbol{=}\mathbf{(C}_{\mathrm{test}}^{\boldsymbol{*}}\mathbf{-}\mathbf{C}_{\mathrm{test}}^{\boldsymbol{*}}\mathbf{W}{}_{\mathbf{o}}\mathbf{P}_{\mathbf{o}}^{\mathbf{T}}\boldsymbol{)}\mathbf{w}_{\mathrm{top}}\mathbf{p}_{\mathrm{top}}^{\mathbf{T}}\mathbf{q}$
17. Go back to step 4 until you have a prediction for every individual in the training set for every model based on the different settings of parameters such as way of pre-processing, principle components used in the base model, number of bins, smoothing factor and number of orthogonal latent variables in the OPLS-DA top model.
18. Based on the prediction of calibration set select the optimal parameters.
19. Go back to step 3 until you have a prediction for every individual using the optimal parameters.
20. Perform OPLS-DA on complete data with equation 7
21. Calculate the VIP using equation 3 and set all $\mathbf{w}_{\mathrm{top}}$ with VIP < 1 to 0.
22. Refold weight vector $\mathbf{w}_{\mathrm{top}}$ into an $\left( M\times F\times F \right)$ **matrix** $\mathbf{W}_{\mathbf{top}}$, when the components $K_{\mathrm{base}}=2$ in the base model.
23. Plot matrix $\mathbf{W}_{\mathbf{top}}$ with red intensity for negative weights and blue intensity for positive weights.

Equations used in the algorithm:

| **(s1)** | **A**  **B** | $\mathbf{m}_{i_{g}}^{T}\mathbf{=}\frac{{\sum_{n=1}^{N_{i_{g}}} \mathbf{X}_{\log_{i_{g}}}}}{N_{i_{g}}}$  $\mathbf{m}^{T}\mathbf{=}\frac{\sum_{g=1}^{G} \sum_{i_{g}\boldsymbol{=1}}^{\boldsymbol{I}_{g}} \mathbf{m}_{i_{g}}^{T}}{\sum_{g=1}^{G} I_{g}}$ |
| --- | --- | --- |
|  | **C** | $\mathbf{D}_{\mathrm{mc}}\mathbf{=}\mathbf{X}_{\log}\boldsymbol{-}\mathbf{1}\mathbf{m}^{T}$ |

| **(s2)** | **A**  **B** | $\mathbf{m}_{i_{1}}^{T}\mathbf{=}\frac{{\sum_{n=1}^{\boldsymbol{N}_{i_{1}}} \mathbf{X}_{\log_{i_{1}}}}}{N_{i_{1}}}$  $\mathbf{m}_{1}^{T}\mathbf{=}\frac{\sum_{i=1}^{\boldsymbol{I}_{\boldsymbol{1}}} \mathbf{m}_{i_{1}}^{T}}{I_{1}}$ |
| --- | --- | --- |
|  | **C** | $\mathbf{D}_{\mathrm{mc}}\mathbf{=}\mathbf{X}_{\log}\boldsymbol{- 1}\mathbf{m}_{1}^{T}$ |

| **(s3)** | **A**  **B** | $\mathbf{m}_{i_{g}}^{T}\mathbf{=}\frac{{\sum_{n=1}^{\boldsymbol{N}_{i_{g}}} \mathbf{X}_{\log_{i_{g}}}}}{{N_{i}}_{g}}$  $\mathbf{D}_{\mathrm{mc}_{i_{g}}}\mathbf{=}\mathbf{X}_{\log_{i_{g}}}\boldsymbol{-}\mathbf{1}_{i_{g}}\mathbf{m}_{i_{g}}^{T}$ |
| --- | --- | --- |
| **(s4)** | **A** | $\mathbf{s}^{\mathbf{T}}\mathbf{=}\sqrt{\frac{\sum_{g=1}^{G} \sum_{i=1}^{I_{g}} var\left( {\mathbf{D}_{\mathbf{mc}}}_{i_{g}} \right)}{\sum_{g=1}^{G} I_{g}}}$ |
|  | **B** | $\mathbf{S= diag}\left( \mathbf{s}^{\mathbf{T}} \right)$ |
|  | **C** | $\mathbf{X}_{\mathbf{cs}}\mathbf{=}\mathbf{D}_{\mathbf{mc}}\mathbf{S}^{\mathbf{-1}}$ |
| **(s5)** | **A** | $\mathbf{s}_{1}^{\mathbf{T}}\mathbf{=}\sqrt{\frac{\sum_{i=1}^{I_{1}} var\left( {\mathbf{D}_{\mathbf{mc}}}_{i_{1}} \right)}{I_{1}}}$ |
|  | **B** | $\mathbf{S}_{1}\mathbf{= diag}\left( \mathbf{s}_{1}^{\mathbf{T}} \right)$ |
|  | **C** | $\mathbf{X}_{\mathbf{cs}}\mathbf{=}\mathbf{D}_{\mathbf{mc}}\mathbf{S}_{1}^{\mathbf{-1}}$ |
| **(s6)** | **A** | $\mathbf{S}_{\boldsymbol{i}_{\boldsymbol{g}}}\mathbf{= diag}\left( \mathbf{s}_{i_{g}}^{\mathbf{T}} \right)$ |
|  | **B** | $\mathbf{X}_{\mathbf{cs}_{\boldsymbol{i}_{\boldsymbol{g}}}}\mathbf{=}{\mathbf{D}_{\mathbf{mc}}}_{i_{g}}\mathbf{S}_{i_{g}}^{\mathbf{-1}}$ |
| **(s7)** | **A** | $\mathbf{X}_{\mathbf{csn}}\mathbf{=}\left[ \begin{matrix} \mathbf{X}_{\mathbf{1}_{\mathbf{1}}}{\mathbf{N}_{\mathbf{1}}}_{\mathbf{1}}^{\mathbf{-1/2}} \\ \boldsymbol{\vdots} \\ \mathbf{X}_{\boldsymbol{I}_{\boldsymbol{G}}}{\mathbf{N}_{\boldsymbol{I}}}_{\boldsymbol{G}}^{\mathbf{-1/2}} \end{matrix} \right]$ |
|  | **B** | $\mathbf{X}_{\mathbf{csn}}\mathbf{=}\mathbf{T}_{\mathbf{base*}}\mathbf{P}_{\mathbf{base*}}^{\mathbf{T}}\mathbf{+E}$ |
|  | **C** | $\mathbf{T}_{\mathbf{base}}\mathbf{=}\mathbf{X}_{\mathbf{cs}}\mathbf{P}_{\mathbf{base*}}$ |
| **(s8)** |  | $\delta_{k}\mathbf{=}\frac{\mathbf{percentile99.95}\left( \mathbf{t}_{k} \right)\boldsymbol{-}\mathbf{percentile0.05}\left( \mathbf{t}_{k} \right)}{F}$ |
| **(s9)** |  | $\mathbf{H}_{i}\boldsymbol{=}\mathbf{H}_{i}\boldsymbol{/}\mathbf{N}_{i}$ |
| **(s10)** |  | ${\mathbf{(I+2}\lambda\mathbf{D}_{\mathbf{1}}^{\mathbf{T}}\mathbf{D}_{\mathbf{1}}\mathbf{+}{\lambda^{2}\mathbf{D}}_{\mathbf{2}}^{\mathbf{T}}\mathbf{D}_{\boldsymbol{2}}\mathbf{) Ĥ}}_{i}\mathbf{=}\mathbf{H}_{i}$ |
| **(2)** |  | $\mathbf{H}= \left[ \begin{matrix} \mathbf{H}_{1,1,1} & \cdots\mathbf{H}_{1,F^{2},1} \cdots& \mathbf{H}_{1,F^{2}, M} \\ \vdots& \ddots\vdots\ddots& \vdots\\ \mathbf{H}_{I,1,1} & \cdots\mathbf{H}_{I,F^{2},1}\cdots& \mathbf{H}_{I, F^{2},M} \end{matrix} \right]$ |
| **(s11)** |  | $\mathbf{H}^{\mathbf{*}}\mathbf{=}\mathbf{t}_{\mathrm{top}}\mathbf{p}_{\mathrm{top}}^{\mathbf{T}}\mathbf{+}\mathbf{T}_{\mathbf{o}}\mathbf{P}_{\mathbf{o}}^{\mathbf{T}} \mathbf{+}\mathbf{E}_{\mathrm{OPLS}}$ |
| **(3)** |  | $\mathrm{VIP}= \sqrt{F^{*}\times\boldsymbol{w}_{top}^{2}}$ |

Supplementary Figure 10: Aliquot 1. a) CD62L versus CD16 before LPS. b) CD62L versus CD16 after LPS. Blue rounds correspond to the cells relatively more present in control individuals (CM), orange crosses correspond to the mature neutrophils (LM) more present in LPS responders and yellow triangles immature neutrophils (LI) according to DAMACY in Figure 1. c) bar plot shows the mean marker expression of each gated group. Although blue rounds overlap with yellow triangles they do have a different expression on the other markers.

Supplementary Figure 9: Aliquot 2. a) CD62L versus CD16 before LPS. b) CD62L versus CD16 after LPS. Blue rounds correspond to the cells relatively more present in control individuals (CM), orange crosses correspond to the mature neutrophils (LM) more present in LPS responders and yellow triangles immature neutrophils (LI) according to DAMACY in Figure 1. c) bar plot shows the mean marker expression of each gated group. Although blue rounds overlap with yellow triangles they do have a different expression on the other markers.

Supplementary Figure 12: Aliquot 3. a) CD62L versus CD16 before LPS. b) CD62L versus CD16 after LPS. Blue rounds correspond to the cells relatively more present in control individuals (CM), orange crosses correspond to the mature neutrophils (LM) more present in LPS responders and yellow triangles immature neutrophils (LI) according to DAMACY in Figure 1. c) bar plot shows the mean marker expression of each gated group. Gating in this aliquot between LI and LM was hard as these two groups partly overlap.


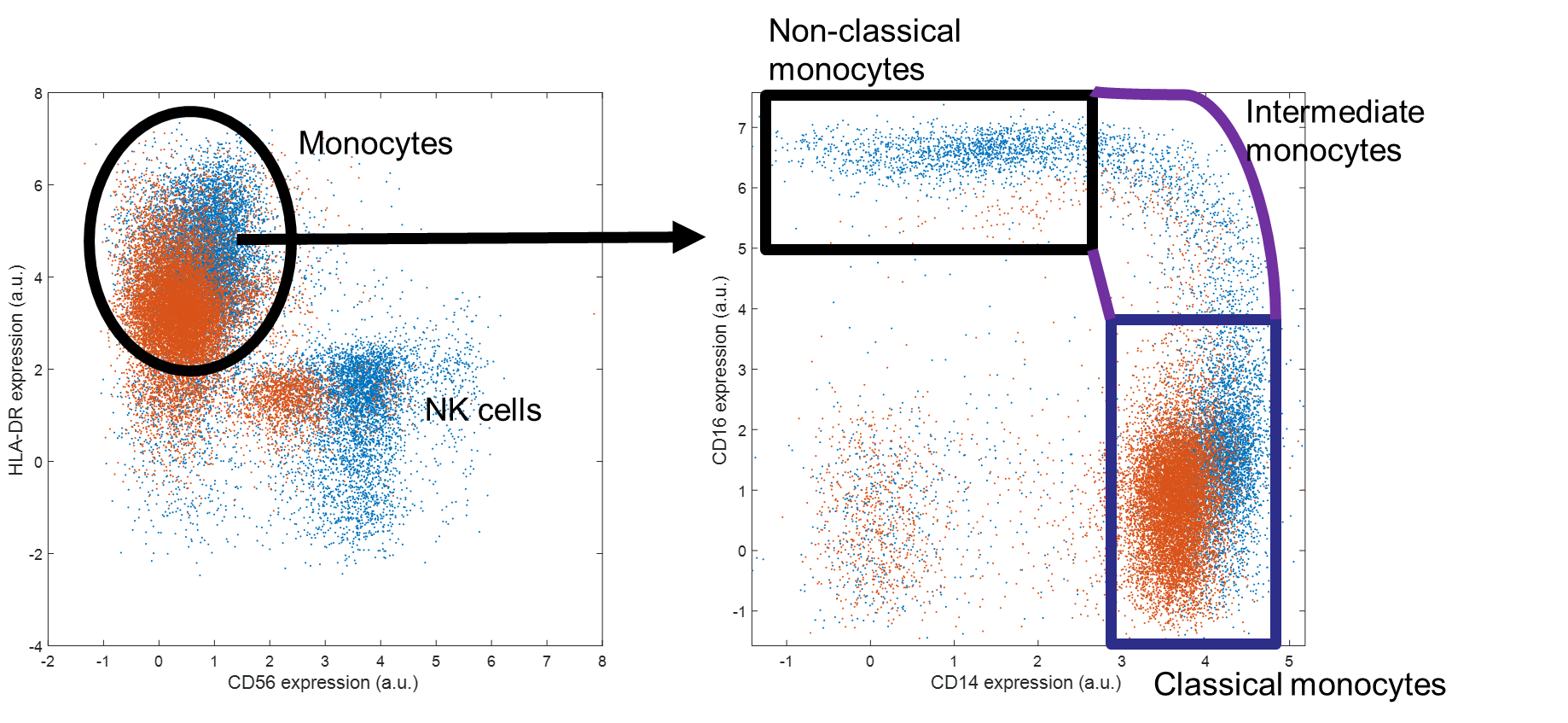


Supplementary Figure 13: Sequential bivariate gating example of obese (blue dots) and lean (orange dots) individuals. Cells positive for CD66bCD3CD19 were removed prior to this Figure and the data was transformed using arcsinh with cofactor 150. Monocytes were first selected by high expression of HLA-DR and low expression of CD56 and subsequently plotting CD14 versus CD16 shows the unique relationship between classical monocytes, intermediate monocytes and non-classical monocytes. Obese individuals show far more non-classical monocytes than lean individuals as was also found with DAMACY (gate I). Not shows here is that the classical monocytes differ on other markers, see gate G (magenta) and H (cyan) below.

# Single marker histograms of B cell dataset

Below, you can see the Figures of the single marker histogram of the gated data in area A (red) and B (blue) as seen in Figure 2, the rest of the cells are plotted in black.

# Single marker histograms of T cell dataset

Below, you can see the Figures of the single marker histogram of the gated data in area C (red) and D (blue) as seen in Figure 2, the rest of the cells are plotted in black.

# Single marker histograms of innate cell dataset

Below, you can see the Figures of the single marker histogram of the gated data in area E (green), F (yellow), G (magenta), H (cyan), I (blue) and J (red) as seen in Figure 2, the rest of the cells are plotted in black.
